# Functions of mucosal associated invariant T cells in eye diseases

**DOI:** 10.3389/fimmu.2024.1341180

**Published:** 2024-02-19

**Authors:** Chihiro Fukui, Satoshi Yamana, Yanqi Xue, Mariko Shirane, Hiroki Tsutsui, Kenichiro Asahara, Keiko Yoshitomi, Takako Ito, Tantri Lestari, Eiichi Hasegawa, Nobuyo Yawata, Atsunobu Takeda, Koh-Hei Sonoda, Kensuke Shibata

**Affiliations:** ^1^Department of Ophthalmology, Graduate School of Medical Sciences, Kyushu University, Fukuoka, Japan; ^2^Department of Ocular Pathology and Imaging Science, Graduate School of Medical Sciences, Kyushu University, Fukuoka, Japan; ^3^Department of Microbiology and Immunology, Graduate School of Medicine, Yamaguchi University, Ube, Japan; ^4^Department of Molecular Immunology, Research Institute for Microbial Diseases, Osaka University, Suita, Japan

**Keywords:** eye, autoimmune disease, metabolite, T cells, immunotherapy

## Abstract

Mucosal-associated invariant T (MAIT) cells are a unique subset of T cells that recognizes metabolites derived from the vitamin B2 biosynthetic pathway. Since the identification of cognate antigens for MAIT cells, knowledge of the functions of MAIT cells in cancer, autoimmunity, and infectious diseases has been rapidly expanding. Recently, MAIT cells have been found to contribute to visual protection against autoimmunity in the eye. The protective functions of MAIT cells are induced by T-cell receptor (TCR)-mediated activation. However, the underlying mechanisms remain unclear. Thus, this mini-review aims to discuss our findings and the complexity of MAIT cell-mediated immune regulation in the eye.

## 1 Introduction

The eye is the organ responsible for visual function, which is associated with quality of life. Therefore, it is essential that harmful immune responses in the eye are strictly regulated. The unique immunoregulatory features of the eye were first recognized by Peter Medawar in the mid-20th century. He showed that tissue from nongenetically identical animals was successfully grafted to the anterior chamber of the eye (1). T cells play a central role in graft rejection. Thus, most studies have focused on understanding how their functions are regulated in the eye. Under normal conditions, the immune-privileged environment in the eye is established through physical compartmentalization by the blood–retinal barrier, which is composed of retinal pigment epithelial cells and retinal vascular endothelial cells (2). The blood–retinal barrier sequesters retinal T-cell antigens within the eye (3). T cells in the eye are not only functionally regulated through TCR-mediated signals upon recognition of their cognate antigens. They are also regulated by other interactions with retinal glial Müller, retinal pigment epithelial, corneal endothelial and ciliary body epithelial cells. These interactions are mediated through inhibitory cell-surface molecules, such as membrane-bound transforming growth factor-β (TGF-β), Fas ligand, cytotoxic T-lymphocyte-associated protein 4, galectin-1 and thrombospondin (4). In addition to cell-contact dependent mechanisms, ocular fluids contain various immunoregulatory molecules such as TGF-β2, interleukin (IL)-10 and a series of neuropeptides. Notably, immunosuppressive factors in the eye lead to systemic tolerance. The administration of foreign antigens to the anterior chamber of the eye induces the migration of antigen-capturing macrophages from the eye to peripheral tissues such as the spleen, thereby dampening antigen-specific inflammatory responses in the periphery (5, 6). This phenomenon is known as anterior chamber-associated immune deviation.

In addition to the eye, the thymus plays a role in maintaining homeostasis by eliminating pathogenic T cells that recognize self-antigens. After T cell progenitors enter the thymus, functional T cells undergo positive selection. Among the selected functional T cell repertoires, most self-antigen-specific T cells are deleted or are functionally energized via negative selection. In this process, self-antigen-presenting medullary thymic epithelial cells dictate T cell fates via high affinity binding with the self-antigen-specific T cells. Arrays of gene expressions that encode tissue-specific self-antigens are regulated by a transcriptional regulator protein, Autoimmune regulator (Aire) (7). However, some self-antigen-specific T cells with potential to cause autoimmunity escape this checkpoint. Autoimmune uveitis is an autoimmune disorder of the eye that is thought to be induced by such escapee T cells. Indeed, using experimental autoimmune uveitis (EAU) models, T cells recognizing self-antigens such as arrestin, interphotoreceptor retinoid-binding protein (IRBP), rhodopsin, recoverin and phosducin have been identified (8). Using EAU mouse models, the breakdown of central tolerance in the thymus was reported to worsen clinical symptoms of EAU, as genetic depletion of the gene encoding the retinal T-cell antigen, IRBP, augmented the antigen-specific T-cell response to IRBP and thereby enhanced ocular inflammation in the retina (9). In support of this, mice lacking the *Aire* gene responsible for expressing tissue-specific self-antigens including IRBP in the thymus spontaneously developed retinal autoimmunity (7, 10). Furthermore, after identification of the regulatory T (Treg) cells responsible for maintenance of immunological self-tolerance and homeostasis (11), accumulating studies have shown that Treg cells play a role in regulating the pathogenesis of autoimmune disorders including autoimmune uveitis (12, 13). Depletion of Treg cells leads to increased susceptibility to EAU (9, 14, 15).

Other T cell subsets, such as natural killer T (NKT) and mucosal-associated invariant (MAIT) cells, have been reported to be involved in ocular immunity through the recognition of nonpeptide antigens such as lipids and metabolites. In an EAU mouse model, NKT cell activation by alpha-galactosylceramide (α-GalCer), a prototype antigen, conferred mitigation of clinical symptoms partly through innate interferon-gamma (IFNγ) production that reduced pathogenic IFNγ and IL-17A production (16, 17). This finding was further supported by a study that showed administration of RCAI-56, a Th1-biased NKT cell ligand, to EAU mice had higher therapeutic efficacy than α-GalCer (18). More recently, protective effects against EAU have been observed upon activation of MAIT cells with the authentic antigen, 5-(2-oxopropylideneamino)-6-D-ribitylaminouracil (5-OP-RU) by IL-22 secretion (19). This data suggest that the inflammatory responses triggered by retinal antigen-specific T cells during EAU induction are counteracted not only by Treg cells but also by other T-cell subsets including NKT and MAIT cells.

Among these T cell subsets involved in ocular immunity, we focus on MAIT cells in the following sections.

## 2 MAIT cells

**Figure 1:**
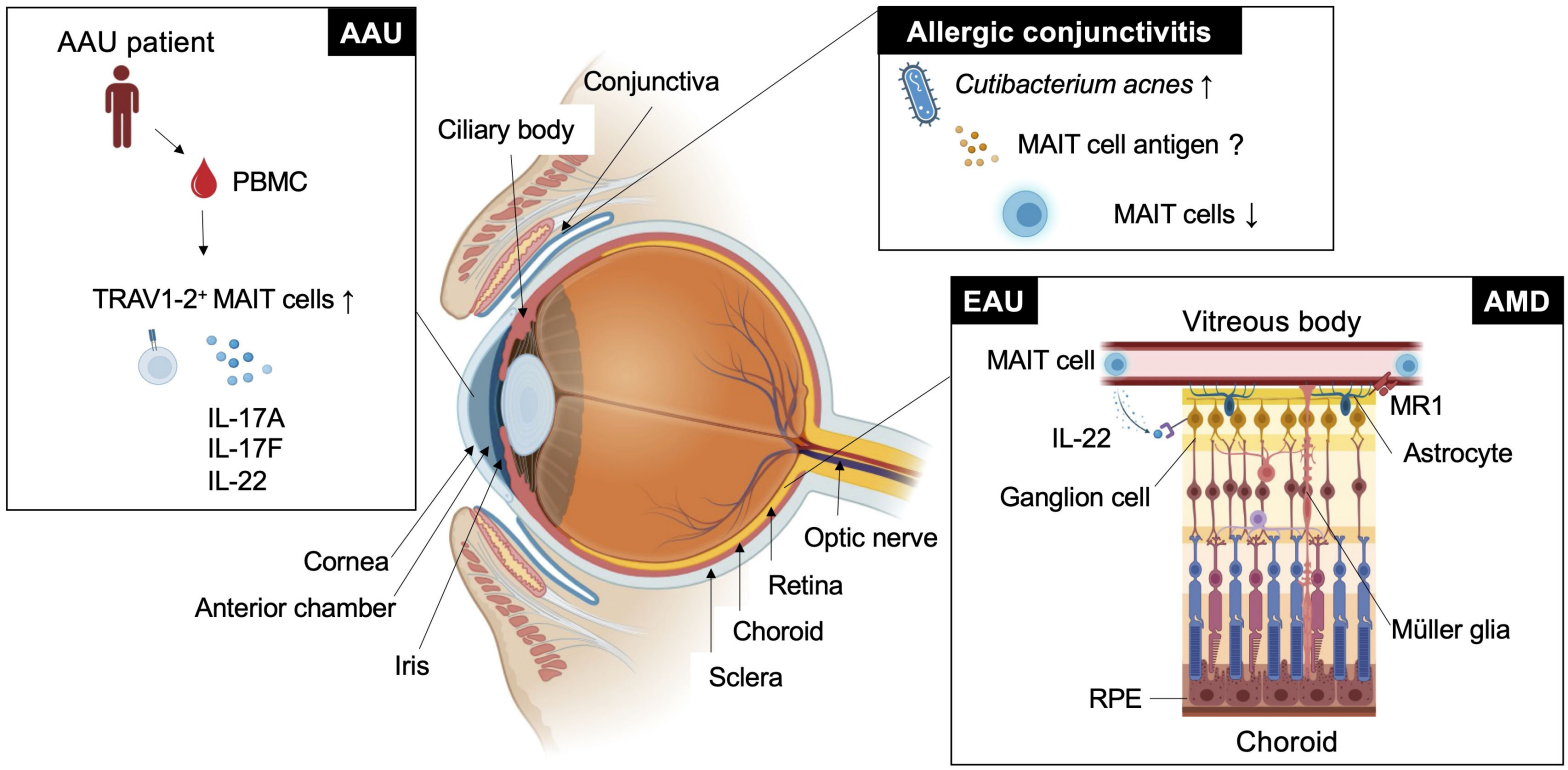
MAIT cell functions in eye diseases. This figure shows how MAIT cells or TRAV1-2^+^ cells are involved in ocular diseases such as experimental autoimmune uveitis (EAU), age-related macular degeneration (AMD), acute anterior uveitis (AAU), and allergic conjunctivitis in different locations.

MAIT cells are a subset of innate-like T cells that require major histocompatibility complex (MHC) class I-related molecule 1 (MR1) for their development (20). MR1 is an antigen-presenting molecule that captures metabolites such as 5-OP-RU and 5-(2-oxoethylideneamino)-6-D-ribitylaminouracil, derived from intermediates in the vitamin B2 biosynthetic pathway, which is present in bacteria and fungi but not in mammals (21, 22). MAIT cells recognize the antigen-MR1 complex through semi-invariant αβ T-cell receptors (TCRs). In humans, these are typically TRAV1-2-TRAJ33/12/20 α chains paired preferentially with limited β chains such as TRBV6-1, TRBV6-4, and TRBV20, and Trav1-Traj33 α chains paired with β chains, such as Trbv13 and Trbv19 in mice. Germ-free mice had a severely reduced MAIT cell number, but monocolonization with vitamin B2-producing bacteria recovered MAIT cell development in the thymus (23–26). These findings suggest that MAIT cells develop or expand in response to 5-OP-RU derived from symbiotic bacteria (26). Consistent with these studies, it has been reported that human MAIT cell frequency is very low from the fetal to perinatal period and rapidly expands after birth when the individual is exposed to symbiotic bacteria (27, 28). Thus, the development and function of MAIT cells are regulated by symbiotic bacteria during early ontogeny. Because this developmental program is evolutionally conserved among mammals, MAIT cells may have nonredundant functions in adult life (29, 30). In support of this, MAIT cells have been shown to play an important role in infection, autoimmunity, and cancer through the production of various inflammatory mediators such as cytokines, cytotoxic molecules and growth factors. MAIT cells were activated after co-culture with bone marrow-derived dendritic cells infected with wide variety of bacteria including *Pseudomonas aeruginosa*, *Klebsiella pneumoniae*, *Lactobacillus acidophilus*, *Staphylococcus aureus* and *Streptococcus epidermidis* (23). Protective roles of MAIT cells have been reported in infection against *Streptococcus pneumoniae* (31), *Klebsiella pneumoniae* (32), *Francisella tularensis* (33) and *Legionella longbeachae* (34). Among these bacteria, *Staphylococcus aureus*, *Streptococcus pneumoniae* and *Pseudomonas aeruginosa* are the major causal agents of eye diseases such as bacterial conjunctivitis, keratitis and endophthalmitis (35), although the involvement of MAIT cells has not been reported. In the tumor environment, MAIT cells have not only cytotoxic activity against tumors (36, 37) but also tumor-promoting activity (38). Recently, a new function of MAIT cells related to tissue homeostasis has been proposed (39). In a wound-healing mouse model after a skin-punch biopsy, MAIT cells had tissue repair functions that were inducible by the administration of MAIT cell antigen 5-OP-RU (25). In the brain, MAIT cells maintain tissue integrity through the production of antioxidant molecules. MAIT cell deficiency in mice accumulates reactive oxygen species around the meninges, impairing meningeal barrier function (40). Such context dependent multifaceted MAIT cell functions raise the question of how they are regulated *in vivo*. MR1-dependent MAIT cell activation is required for host defense against *Legionella* and *Francisella* infection (34, 41, 42). In contrast, in a wound-healing mouse model, the migration and tissue repair function of MAIT cells were MR1-independent (43). These findings have been summarized in previous review articles (44–47). However, in these articles, the role of MAIT cells in the eye has not been well described. Nevertheless, association of MAIT cell frequencies in particular autoimmune diseases such as ankylosing spondylitis and Sjogren syndrome with ocular manifestations suggests the potential role in the eye (48–50). Therefore, we aimed to introduce the role of MAIT cells in autoimmune uveitis and further discuss other eye diseases, such as age-related macular degeneration (AMD), allergic conjunctivitis and acute anterior uveitis (AAU), in which MAIT cells may have an immunoregulatory role (**Figure 1**).

## 3 MAIT cells and eye diseases

### 3.1 MAIT cells in autoimmune uveitis

**Figure 2:**
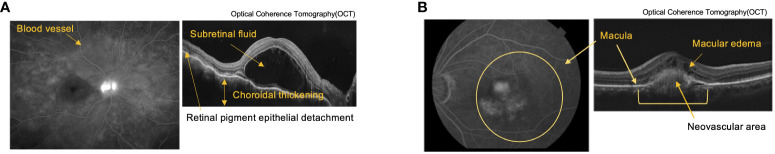
Pathological tissue images of autoimmune uveitis and Age-related Macular Degeneration (AMD). **(A)** Fluorescein Angiography (FA) (left) and Optical Coherence Tomography (OCT) (right) images of Vogt-Koyanagi-Harada (VKH) disease. FA shows multiple fluorescent leaks around the blood vessel. OCT shows retina layers with subretinal fluid, choroidal thickening, and retinal pigment detachment. **(B)** Indocyanine Green Angiography (left) and OCT (right) images of AMD. Images shows layers of retina with choroidal neovascularization associated with macular edema due to exudative changes.

Autoimmune uveitis is a leading cause of blindness among patients with uveitis in the United States and Asia (51, 52). Autoimmune uveitis has two types: eye-specific inflammation, such as Sympathetic ophthalmia and Birdshot retinochoroidopathy, and systemic inflammation that also affects the eye, such as Behcet’s disease, Sarcoidosis and Vogt-Koyanagi Harada (VKH) disease (4). In these patients, severe inflammation is frequently observed in the retina, located in the posterior compartment of the eye where neural and photoreceptor cells responsible for the transmission of visual information to the brain are present (**Figure 1** and **Figure 2A**). Recognition of retinal antigens by T cells is thought to induce chronic inflammation in the retina and adjacent structures including optic nerves, leading to impairment of visual functions resulting from cell damage. In contrast to the pathogenic roles of T cells, their protective functions remain less understood. Dr. R. R. Caspi’s group showed the protective functions of IL-22-producing T cells using a mouse model of EAU induced by the retinal antigen IRBP (53). IL-22 bound to the IL-22 receptor on retinal ganglion cells and prevented their cell death (53) (**Figure 1**). Another possible protective mechanism could be that IL-22 can suppress MHC class II expression responsible for the development of retinal antigen-specific T cells (54). MAIT cell frequency in the peripheral blood has been found to be inversely correlated with disease activity in patients with VKH disease, which is representative of autoimmune uveitis with chronic inflammation in the retina (19). These findings motivated us to explore MAIT cell functions in a mouse model of EAU induced by the retinal antigen IRBP. MAIT cells also secrete IL-22 and contribute to the reduction of EAU clinical symptoms after EAU induction (19). MAIT cells were hardly detected in the retina under normal conditions and gradually increased after EAU induction. This finding suggests that MAIT cells migrate from the draining lymph nodes, where preceding MAIT cell expansion was observed (19). The involvement of TCR-mediated signaling in MAIT cell expansion during EAU induction has not been experimentally proven. However, TCR-mediated activation of MAIT cells was observed in the eyes and draining lymph nodes of EAU-induced mice. The MAIT TCR-mediated signal has a therapeutic potential. Administration of the cognate antigen 5-OP-RU improved clinical symptoms and visual function, although the underlying mechanism need to be further explored (19).

Previous studies have implicated TCR-mediated MAIT cell activation by the gut microbiota in EAU. *Bacteroides* and *Parabacteroides* species belonging to *Bacteroidetes* were significantly increased in patients with VKH disease compared with healthy controls (55), and *Bacteroidetes* contained species that produce metabolites with higher MAIT cell agonistic activity (56). The activation of MAIT cells by metabolites from symbiotic bacteria can occur in the eyes of individuals with autoimmune uveitis, as 5-OP-RU has been reported to travel between distal organs (26). Furthermore, MAIT cells may be primed in the gut and infiltrate the eye, as in the case of retina-specific T cells (57). However, a more precise analysis to investigate how and where MAIT cell functions are regulated *in vivo* is required.

### 3.2 Implication of the immunoregulatory functions of MAIT cells in other eye diseases

AMD is a neurodegenerative disorder with a similar pathogenicity to Alzheimer’s and Parkinson’s disease. The similarity in anatomy and cellular composition between the retina and brain prompted the consideration of similar immunological roles of MAIT cells. AMD causes progressive photoreceptor degeneration in the macula, leading to vision loss alongside with aging (**Figure 2B**). In a previous study, single-cell RNA sequencing analysis using human retinal cells demonstrated that the expression of AMD-associated genes identified by a genome-wide association study (GWAS) was highly biased toward particular retinal cell populations, including Müller glia and astrocytes (58). Another study showed that the highest expression of *MR1* was observed in the retinal astrocytes of patients with AMD (59) (**Figure 1**). Astrocytes are resident neural cells present in the brain and retina. Brain astrocytes have been shown to express MR1 and have the potential to activate MAIT cells (60). MAIT cells have been reported to play a protective role in neuroinflammation by maintaining tissue integrity through the productions of IL-10 and antioxidative molecules in the brain (40, 61). However, MAIT cell functions in AMD remain unclear.

The conjunctiva is a mucosal tissue in the anterior eye with barrier functions against pathogenic insults. These functions are mediated by resident immune cells, and dysregulated activation of immune cells can cause allergic conjunctivitis. Allergic conjunctivitis is associated with chronic inflammation at the ocular surface where there is immunoregulatory interplay between commensal microbiota, conjunctival intraepithelial lymphocytes and epithelial cells (62). Approximately 16% of CD45^+^ leukocytes in the upper tarsal conjunctiva of healthy individuals were MAIT cells (63). MAIT cell frequency was not increased at the ocular surface in patients with chronic allergic conjunctivitis, in whom *Cutibacterium acnes* became the predominant commensal bacterial population (63) (**Figure 1**). This suggests that *C. acnes* does not induce MAIT cell expansion as observed in human volunteers infected with *Salmonella* Paratyphi A (64). Although *C. acnes* has a vitamin B2 synthetic pathway, it has a low MAIT cell-stimulating ability (56). Thus, it is possible that *C. acnes* has evolved to escape adaptive immunity by reducing MAIT cell antigen production, as described in *Salmonella* and *Francisella* sp. (41, 42). Thus, examining whether MAIT cell functions are regulated by *C. acnes* in allergic conjunctivitis in future studies would be interesting.

AAU is the most common form of uveitis (65) and frequently accompanies HLA-B27-related inflammatory diseases, such as ankylosing spondylitis and spondyloarthropathies (66). AAU manifests as an acute onset of nongranulomatous uveitis, characterized by cellular and protein extravasation into the aqueous humor. GWAS between patients with AAU and healthy donors using peripheral blood revealed several AAU-associated loci, including *IL6R*, *IL10*, *IL19* and *IL18R* (67). IL-18R is one of the authentic markers for MAIT cells (23). The frequency of MAIT cell-enriched TRAV1-2^+^ cells producing IL-17A, IL-17F and IL-22 was increased in the peripheral blood compared with the healthy control (68) (**Figure 1**). It remains to be determined whether enrichment of immunoregulatory MAIT cells also occurs in the anterior chamber of the eye.

## 4 Discussion for future direction

The retina converts light stimuli into signals and transmits visual images to the brain. Layered nerve cell populations transmit signals one after another through intercellular interactions to accomplish their complex functions, thus maintaining visual function (**Figure 1**). Degeneration or dysfunction of only one of the component cells can destroy this sophisticated interaction (69). Under inflammatory conditions, infiltrating inflammatory cells, such as monocytes, macrophages, and T cells, and resident glial cells, such as astrocytes and microglial cells, have immunoregulatory functions to maintain tissue homeostasis in the retina and uvea. Therefore, understanding spatiotemporal cell functions in the eye is essential for understanding the pathogenesis of certain diseases. Recent advances in single-cell technology with spatial information allows us to understand their functions and mutual interactions at the single-cell level. Elucidation of the regulatory network could open a new avenue to clarify the full picture of ocular immunity and how MAIT cells are involved in this process.

Accumulating evidence has shown that MAIT cells play an immunoregulatory role in autoimmune diseases targeting various organs, including the eye. In the case of autoimmune uveitis, recent findings on the therapeutic potential of TCR-mediated MAIT cell activation have motivated us to identify putative MAIT cell antigens in EAU mice. More detailed analysis of metabolites using highly sensitive mass spectrometry (70–72) and single metabolite-probing technology (73) will allow us to test this possibility.

## Author contributions

CF: Writing – review & editing. SY: Writing – review & editing. YX: Writing – review & editing. MS: Writing – review & editing. HT: Writing – review & editing. KA: Writing – review & editing. KY: Writing – review & editing. TI: Writing – review & editing. TL: Writing – review & editing. EH: Writing – review & editing. NY: Writing – review & editing. AT: Writing – review & editing. KHS: Supervision, Writing – review & editing. KS: Conceptualization, Funding acquisition, Writing – original draft, Writing – review & editing.
